# Biogeographical ‍distributions of trickster animals

**DOI:** 10.1098/rsos.231577

**Published:** 2024-05-08

**Authors:** Shota Shibasaki, Ryosuke Nakadai, Yo Nakawake

**Affiliations:** ^1^ Department of Biology, The University of North Carolina at Greensboro, Greensboro, NC, 27412, USA; ^2^ Center for Frontier Research, National Institute of Genetics, Mishima, Shizuoka, 411-8540, Japan; ^3^ Biodiversity Division, National Institute for Environmental Studies, Tsukuba, Ibaraki, 305-8506, Japan; ^4^ Faculty of Environment and Information Sciences, Yokohama National University, Yokohama, Kanagawa, 240-8501, Japan; ^5^ Institute for Multidisciplinary Sciences, Yokohama National University, Yokohama, Kanagawa, 240-8501, Japan; ^6^ Department of Social Psychology, Yasuda Women’s University, Asaminami-ku, Hiroshima, 731-0153, Japan; ^7^ School of Anthropology and Museum Ethnography, University of Oxford, Oxford, OX2 6PE, UK

**Keywords:** folklore, cultural evolution, species distribution, biome, ecological constraints

## Abstract

Human language encompasses almost endless potential for meaning, and folklore can theoretically incorporate themes beyond time and space. However, actual distributions of the themes are not always universal and their constraints remain unclear. Here, we specifically focused on zoological folklore and aimed to reveal what restricts the distribution of trickster animals in folklore. We applied the biogeographical methodology to 16 taxonomic categories of trickster (455 data) and real (93 090 848 data) animals obtained from large databases. Our analysis revealed that the distribution of trickster animals was restricted by their presence in the vicinity and, more importantly, the presence of their corresponding real animals. Given that the distributions of real animals are restricted by the annual mean temperature and annual precipitation, these climatic conditions indirectly affect the distribution of trickster animals. Our study, applying biogeographical methods to culture, paves the way to a deeper understanding of the interactions between ecology and culture.

## 1. Introduction

The hallmark of human language communication is its expressibility. It can enable us to communicate topics remotely in time and space (i.e. displacement [1]). Folklore is an aspect of human culture that strongly reflects the expressive characteristics of human language. In theory, folklore can refer to animals unseen by storytellers and even describe imaginary animals that do not exist in the real world [2]. Such fictional features can stimulate our curiosity and explorative tendencies [3]. However, worlds invented for fiction are not free from cognitive constraints. For example, the cost of a magical spell that violates physical laws is not randomly decided; rather, it is based on actual inferences about the physical world [4]. Similarly, ecological factors can restrict the content of folklore. This study focuses on the ecological factors that restrict the theoretically infinite meaning spaces of folklore.

Researchers have discussed the relationship between cultural and ecological factors for decades. Anthropologists, geographers and other social science and humanities scholars have argued that natural environments are a major source of cultural diversity [5,6]; for example, material cultural artefacts such as hunting tools vary across environments [7,8]. In addition, the environment can affect non-material cultures. Recent studies show that climatic and/or ecological factors affect political ideologies [9], individualism and collectivism [10], social trust [11], belief in moralizing gods [12,13] and faith in giant trees [14].

Commonly perceived as a collection of traditional stories that transmit cultural identity among social groups, folklore (detailed definition in §2.1) is an example of non-material cultures affected by the environment. Folklore is also vital in acquiring ecological knowledge of the local environment [15–17]; for instance, the folk-biological knowledge or locals’ understanding of harmful animals [18], and the pairing of wild and domestic animals in antagonistic interactions [19].

Biogeography has, for decades, delved into the determinants of species distribution in nature [20]. Climate conditions are predominant among the numerous biotic and abiotic factors affecting species distributions. For example, many studies have reported shifts in animal and plant distributions owing to climate change [21–24]. The concept of biomes, or units of plant assemblages and associated animal species, highlights the importance of climate conditions on species distributions [25–27]; thus, biomes worldwide are classified based on climate conditions [28,29]. One of the most famous biome concepts was proposed by Whittaker [30]. Whittaker’s biome classifies the environment into nine (plus one as an outlier) biomes based on annual mean temperature and annual precipitation.

Do animal distributions in folklore reflect the climatic conditions and distributions of real animals? This non-trivial question remains under-researched. Theoretically, folklore can contain any creatures regardless of the local environment due to the expressibility of human language. For example, folklore concerning imaginary animals such as dragons exists worldwide [2,31,32], even though such creatures do not exist in the real world. Folklore of some real carnivores remains in regions where these animals have gone extinct, e.g. bears in Britain [33,34] and wolves in Japan [35]. The distribution of real and fictional animals should be mismatched if motifs of fictional animals are transmitted freely across ecological conditions. However, ecological conditions are likely to restrict the animal distribution in folklore because folklore contains the ecological knowledge of local environments [15–17].

**Figure 1.**
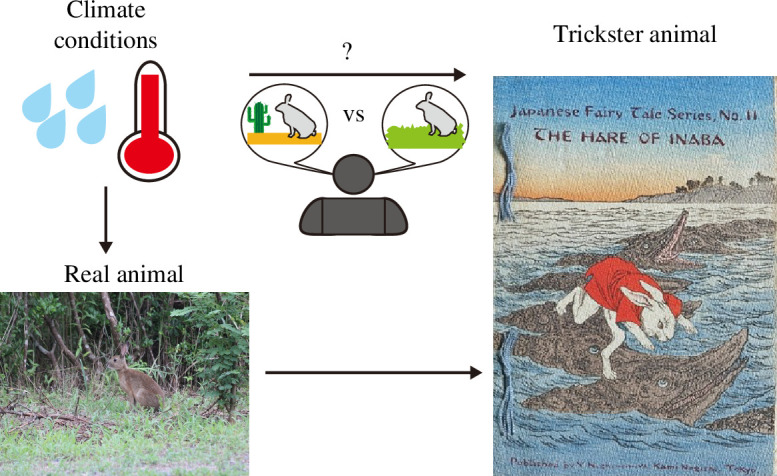
Constraints on the trickster animal distributions. Schematic representations of the manuscript show two environmental conditions: annual mean temperature and annual precipitation. These attributes affect the distribution of real animals that would potentially be represented as tricksters. The distribution of real animals denotes a necessary condition for the presence of corresponding trickster animals. This one presents the Japanese hare, *Lepus brachyurus* (Photo by Dr Abby Darrah https://www.inaturalist.org/observations/105058298, CC-BY), and ‘The Hare of Inaba’ (Illustration by Eitaku Kobayashi) as examples of real and trickster hare, respectively. The image of ‘The Hare of Inaba’ was obtained from the library of the Open University of Japan.

Here, we statistically analysed databases on folklore, real animals and climate conditions to find the determinants of animal distribution in folklore (figure 1). We used motifs of animal or zoomorphic tricksters, characters performing tricks, because they are stable and worldwide motifs [36–39]; see §2.1 for a more detailed definition. Berezkin’s collection has accumulated various types of folkloristic motifs worldwide, including trickster animals [36], with geographic coordinate data where folklore was recorded. This provides an ideal opportunity to quantitatively analyse the distribution of trickster animals. We hypothesized that (i) climate conditions regulate animal distribution in folklore as in nature and (ii) there is an overlap in the distributions of real and trickster animals in folklore. To test these hypotheses, we classified the climate conditions where tricksters and/or real animals were sampled into Whittaker’s biomes [30]. We compared the fractions of the biomes in real and trickster animals and found that the distributions of real animals were restricted by climate conditions and that the presence of real animals restricted the distributions of trickster animals. In other words, climate conditions indirectly restrict the distribution of trickster animals in folklore. These results suggest that ecological factors could restrict the contents of folklore or, more broadly, human culture, due to human cognitive biases.

## 2. Methods

### 2.1. Definitions of folklore, motif and trickster

This subsection describes folklore and details the motif of tricksters in folklore. The term ‘folklore’ can include material cultures [40] but commonly refers to oral traditions. Bascom [41] defined folklore as prose narratives including three categories: folktales, legends and myths. We use an operational definition of folklore in this study as any records incorporated in the lifelong work of Dr Yuri Berezkin, The Thematic Classification and Areal Distribution of Folklore-Mythological Catalogue [42,43].

The catalogue includes more than 3000 motif indexes developed by Berezkin, who defined motifs as ‘any episodes or images retold or described in narratives that are registered in at least in two (although normally in many more) different traditions’ [43, p. 37]. Berezkin classified motifs into 13 major categories, labelling them with letters from A to N; among such motifs, themes incorporating tricksters are classified as ‘M: ПРИКЛЮЧЕНИЯ III: ПРОДЕЛКИ И ЭПИЗОДЫ (M. Adventures III: Mischief and Episodes; translated by authors; see https://www.ruthenia.ru/folklore/berezkin/)’. We used this catalogue for two reasons. First, Berezkin’s catalogue includes worldwide folklore [44], enabling us to compare distributions of real and trickster animals globally. Second, this catalogue provides geographic coordinate data of folklore, which enables us to compare the distribution of tricksters and real animals. This is unique to Brezkin’s catalogue because the Aarne Thompson Uther catalogue, which is used in previous studies [19,45], does not provide such geographic data. However, there are some drawbacks of Berezkin’s catalogue. Sources of the database are mainly based on literature written in English, Russian, Spanish, German and French [46]. In addition, Berezkin’s catalogue does not contain a motif for broader animal-related tales such as ‘animal tales’, which the Aarne Thompson Uther catalogue contains.

Instead of analysing broad animal folklore, we analysed the motifs of tricksters because animal or zoomorphic tricksters are found worldwide and have stable characteristics [36]. Tricksters are a type of fictional character that performs tricks and deceptions or exhibits mischievous behaviours (e.g. stealing and cheating). The trickster’s role is often metaphorically understood: for instance, as ‘a boundary-crosser’ who travels between or connects two different worlds [47]. Berezkin defined the trickster as ‘any personage who deceives others, acts in a strange way or gets into comical situations but as one who combines two pairs of opposite characteristics which in the norm are related to different types of action’ [48, p. 126].

### 2.2. Data collection

We compiled data on the distributions of trickster animals from Dr Berenzkin’s World Myth database [42,43], real animals from the Global Biodiversity Information Facility (GBIF) [49] and climate conditions from WorldClim 2.1 [50]. We obtained folklore data via personal communication with Dr Yuri Berezkin, downloading it from his database in July 2022. We used the motifs ‘Trickster–*X*’ [m29a–m29i] and ‘Trickster is a(n) *X*’ [m29l–m29y]. The items encased in square brackets show Berezkin’s motif index and *X* represents common animal names. We analysed motifs that satisfied the following three criteria to proceed with the further analysis:

1. The scientific names can be estimated from the focal animals’ common names because scientific names were needed to obtain the animal distribution from GBIF.
2. Grouping multiple animal names together was allowed when it was taxonomically reasonable.
3. Assembling multiple animals was avoided when their distribution was known to be geographically distinct; otherwise, the distribution of trickster and real animals would be biased to overlap more.

In this study, the following 16 categories satisfied the above criteria and were analysed: anteater [m29qq], badger [m29x1], ground squirrel [m29nn], hawk [m29i], mink [m29d], mouse [m29n], opossum [m29l], owl [m29h], porcupine [m29r], rabbit/hare [m29g], raccoon [m29q], rat [m29m], raven/crow [m29a], skunk [m29c], spider [m29p] and wren [m29y]. We removed six motifs from the analysis because they did not satisfy either of the three criteria: (i) monkeys [m29o], (ii) water birds [m29j], (iii) foxes, coyotes, or jackals [m29b], (iv) felines (jaguars, ocelots, or pumas) [m29w], (v) small ungulates [m29v], and (vi) turtles, toads or frogs [m29k].

For example, the types of animals to be included in water birds [m29j] and small ungulates [m29v] were unclear (not satisfying criterion 1), and we could not specify the scientific names of species corresponding to these animals. Similarly, we could not proceed with the analysis of monkeys [m29o] because what ‘monkey’ includes changes over time, and this category can be vaguely used (e.g. whether monkeys include apes or not); see Oxford English Dictionary for details. Grouping turtles, toads and frogs together [m29k] is biologically unreasonable (not satisfying criterion 2) as turtles are reptiles while toads and frogs are amphibians. Foxes, coyotes or jackals [m29b] should be subdivided because the previous study shows that the geographic distribution of their corresponding trickster animals does not overlap (not satisfying criterion 3) [36]. Felines [m29w] include many species whose geographic distributions are distinct (not satisfying criterion 3) [51]. Because the details of these folklores were unavailable, we could not subdivide these data and removed them from further analyses. The amount of data remaining for each trickster animal ranged from 6 to 190 (a total of 455 pieces of data) depending on the category.

We used Wikipedia to assign the scientific names of the corresponding real animals for each trickster animal. We confirmed whether these suggested scientific names matched the common names of the animals by accessing the National Center for Biotechnology Information and the Encyclopedia of Life using the sci2comm() function in the taxize library [52] version 0.9.98 in R (version 4.2.1). Four scientific names (two ground squirrels: *Geosciurus* and *Euxerus*, and two badgers: *Arctonyx hoevenii* and *Melogale subaurantiaca*) did not appear on either database, and we removed these species from further analysis (see also electronic supplementary material). The distributions of the real animals were collected from GBIF using the occ_download function in the rgbif library version 3.7.3 [53] in R. The coordinate data were cleaned using the clean_coordinates function of the CoordinateCleaner library [54] with tests of capitals, centroids, gbif, institutions and zeros. After data cleaning, the data of real animals varied from 5400 to 50 000 000 (a total of 93 090 848 pieces of data) depending on the animal category.

The intensity of data collection relating to tricksters and real animals would probably differ across species and locations. Therefore, we converted the coordinate data into hex grid indices using the geo_to_h3 function in the h3 package version 3.7.4 [55] of Python 3 (version 3.8.13). The resolution of the hex grid is crucial in our analysis. This parameter determines the number of grids where the tricksters and/or real animals exist. Because the number of trickster data pieces is small, enhancing the resolution parameter would increase the statistical power. Meanwhile, the climate conditions may be unavailable with the higher resolution, and the computational costs of the analyses increase over the resolution. We set the resolution of the hex grids = 1, generating 842 grids across the world map, because the number of grids is larger than the number of trickster data pieces and because the climate data (see below) are assigned to almost all grids. Electronic supplementary material, table S1 shows that the number of grids where the presence of the tricksters was reported little changed when the resolution parameter is two or higher. In the electronic supplementary material, we show the results with the resolution of the hex grid = 2 (5882 grids across the world), but these analyses show qualitatively similar results to the main text (electronic supplementary material, tables S2–S4). We did not consider the number of reports per grid in this manuscript; we used only the presence data of the tricksters and real animals in each grid to minimize the effect of sampling biases across species and space. After the data conversion, we obtained 257 pieces of presence data of trickster animals on the hex grids and 3413 pieces of presence data of real animals on the hex grids.

The climate data were assigned to each hex grid after the coordinates of tricksters and real animals had been converted. We retrieved the annual mean temperature and annual precipitation of the centre point of each grid from WorldClim 2.1 [50] using the latlon-utils package version 0.07 [56] in Python 3. We selected data on these two climate conditions because they classify the environment into Whittaker’s biomes [30]. If the annual mean temperature and/or annual precipitation were unavailable (e.g. when a centre point of a grid existed on an ocean), we estimated the two groups of environmental data from the means at the coordinates inside the grid at which real animals were reported. We grouped the data into biome classes using the plotbiomes library [57] in R.

### 2.3. Statistical analyses

We first investigated the fractions of Whittaker’s biome classes. For each animal category, we compared the fractions of the biome classes between the tricksters and corresponding real animals. Furthermore, we compared the fractions of the biome classes with a null model generated by the hex grids and corresponding environmental conditions where at least one of the real animals in our analysis was reported. This null model represents the fractions of the biome classes in terrestrial areas. We used the chi-squared test in R to compare the fractions of the biome classes. We corrected the obtained *p*-values using the false discovery rate (FDR) method with the p.adjust function.

We then investigated whether the presence of tricksters in each grid was limited by the presence of the corresponding real animals. We calculated the conditional probabilities that the corresponding real animals were reported in a grid within which the focal animals appeared as tricksters in folklore. This conditional probability represents whether the corresponding real animals regulate the presence of trickster animals. A very low conditional probability would imply that trickster folklore could be transmitted to areas in which the locals were unfamiliar with the focal animals. Conversely, a high conditional probability would suggest that the presence of real animals was a necessary condition for the presence of trickster animals in the folklore. Notably, this conditional probability did not intend to show the predictability of the presence of trickster animals, which is beyond the scope of this study.

**Figure 2.**
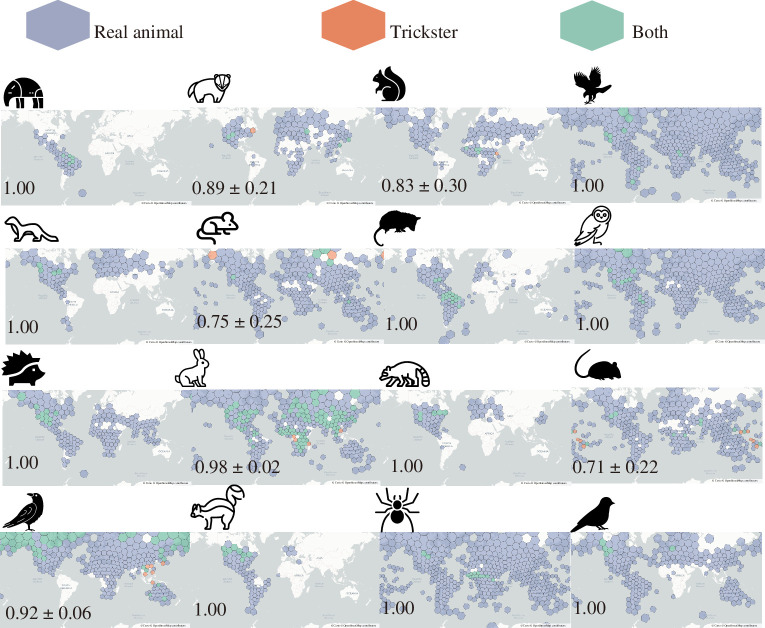
The distribution of real and trickster animals on the world map. The distributions of 16 real and trickster animals (shown by icons) are shown on the world map. The blue, orange and green hex grids, respectively, represent where only the real animals, only the trickster animals or both versions were reported, respectively. The numbers at the bottom left indicate the conditional probabilities that the corresponding real animals existed in the grid where the trickster animals were reported, and their 95% confidence intervals. The enlarged figures are available in electronic supplementary material, figures S1–S16.

Next, we performed a permutation test to determine whether the distribution of each trickster animal was clogged. The above analysis indicated that the presence of the corresponding real animals was necessary for the presence of a trickster in the folklore (figure 2). Therefore, the null hypothesis was postulated—a focal animal appears as a trickster where the corresponding real animals are observed. We compared the median distance between the hex grids where the focal animals were reported as tricksters and the median of the simulated distances under the null hypothesis. The distributions of trickster animals under the null hypothesis were generated by randomly selecting the hex grids within which the corresponding real animals existed, and the number of selected grids was identical to the number of grids that the focal trickster animals were reported. We generated 10 000 such distributions for each animal and obtained the probability distributions of the median distances according to the null hypothesis, which enabled us to calculate *p*-values. The attained *p*-values were corrected by the FDR method using the multitest.fdrcorrection function in the statsmodels library [58] in Python 3.

## 3. Results

### 3.1. Environmental constraints on animal distributions

**Figure 3.**
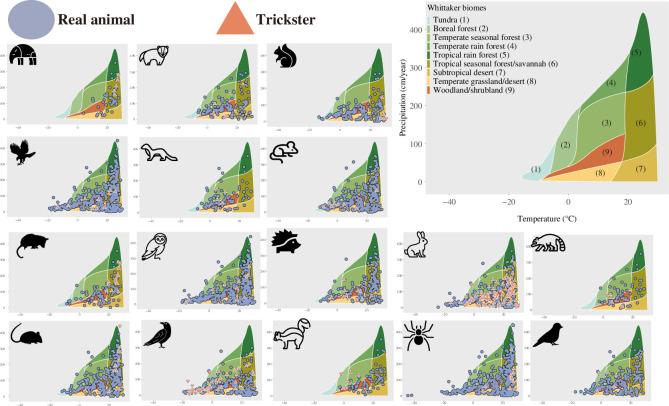
The distribution of real and trickster animals in Whittaker’s biome. The distributions of 16 real and trickster animals (shown by icons) are shown on Whittaker’s biome (top right). The blue circles and the orange triangles in Whittaker’s biome depict the climate conditions of the regions where the real animals and tricksters were reported, respectively. The background colours and the numbers in parentheses represent the biome classes (see the top right panel). The enlarged figures are available in electronic supplementary material, figures S1–S16.

We investigated the effects exerted by climate conditions on the distributions of real and trickster animals (figure 3). We classified climate conditions into nine groups (and one as an outlier) as per Whittaker’s biome classes [30] and compared the fractions of the biome classes between each category of animal and terrestrial areas (i.e. the null model). The second column of table 1 shows that the distributions of 12 of the 16 real animals differ from the null model, suggesting that annual mean temperature and annual precipitation restrict the distribution of many animals. The exceptional animals (i.e. hawk, owl, rabbit or hare and spider) were found on all continents except Antarctica. In contrast, only four animals (mink, opossum, rave or crow, and skunk) differed in the fractions of biome classes between the tricksters and the null model (the third column of table 1). Trickster minks were found in temperate seasonal forests, opossums were noted in tropical seasonal forests/savannahs, ravens or crows were observed in the tundra, boreal forests, template seasonal forests or tropical seasonal forests/savannahs, and skunks were seen in boreal forests or temperate seasonal forests. These analyses provide evidence that annual mean temperature and annual precipitation restrict real animal distributions; however, such environmental constraints are less evident on trickster animal distributions. This may, however, be due to differences in the amounts of data (see §2.2). The quantity of trickster-related data with the grid resolution parameter =1 may be too small (between 4 and 99 pieces of data depending on the animal category, see electronic supplementary material, table S1) in comparison with the number of biome classes (totalling 10); thus, the statistical power may not be large enough. Indeed, this result was sensitive to the resolution parameter; increasing the resolution of the grids shows that fractions of tricksters’ biomes are different from the null model in 12 animal categories (the middle column of electronic supplementary material, table S2) because of the increase in the amount of trickster’s data.

**Table 1.** *P*-values in chi-squared test to compare the frequencies of the biome classes.

category	real versus null	trickster versus null	real versus trickster
anteater	$1.46\times 10^{-4}$ ^a^	$5.00\times 10^{-1}$	$8.26\times 10^{-1}$
badger	$2.13\times 10^{-5}$ ^a^	$1.02\times 10^{-1}$	$5.47\times 10^{-1}$
ground squirrel	$2.09\times 10^{-7}$ ^a^	$5.00\times 10^{-1}$	$2.13\times 10^{-1}$
hawk	$9.96\times 10^{-1}$	$6.29\times 10^{-1}$	$7.55\times 10^{-1}$
mink	$2.59\times 10^{-9}$ ^a^	$4.08\times 10^{-2}$ ^a^	$5.72\times 10^{-1}$
mouse	$1.77\times 10^{-2}$ ^a^	$7.11\times 10^{-2}$	$9.78\times 10^{-4}$ ^a^
opossum	$1.07\times 10^{-2}$ ^a^	$4.08\times 10^{-2}$ ^a^	$1.80\times 10^{-1}$
owl	$9.96\times 10^{-1}$	$8.47\times 10^{-1}$	$7.55\times 10^{-1}$
porcupine	$3.38\times 10^{-2}$ ^a^	$2.45\times 10^{-1}$	$2.18\times 10^{-1}$
rabbit/hare	$8.00\times 10^{-2}$	$7.99\times 10^{-2}$	$2.92\times 10^{-1}$
raccoon	$3.56\times 10^{-7}$ ^a^	$3.45\times 10^{-1}$	$7.69\times 10^{-1}$
rat	$2.99\times 10^{-4}$ ^a^	$5.00\times 10^{-1}$	$5.47\times 10^{-1}$
raven/crow	$2.49\times 10^{-8}$ ^a^	$1.55\times 10^{-7}$ ^a^	$1.81\times 10^{-5}$ ^a^
skunk	$6.45\times 10^{-3}$ ^a^	$4.08\times 10^{-2}$ ^a^	$4.51\times 10^{-4}$ ^a^
spider	$9.96\times 10^{-1}$	$6.29\times 10^{-1}$	$7.55\times 10^{-1}$
wren	$8.84\times 10^{-7}$ ^a^	$3.40\times 10^{-1}$	$5.44\times 10^{-1}$

^a^ *p*-value < 0.05 after FDR correction.

### 3.2. Ecological constraints on animal tricksters

Next, we determined whether the trickster animals were freely distributed across the world or whether their presence was restricted by the presence of their corresponding real animals. For this purpose, we calculated the conditional probability that a corresponding real animal existed in the region where the trickster animal appeared in local folklore. The values in figure 2 show that the conditional probabilities of 14 animals were greater than 80%, suggesting that the presence of real animals is an almost necessary condition for the presence of trickster animals. Qualitatively similar results were obtained when we increased the resolution of the hex grids (electronic supplementary material, table S3). As the real animal distributions were restricted by the two climate conditions, we concluded that these conditions indirectly restricted the distribution of the trickster animals. Further constraints were unclear because only three trickster animals (i.e. mouse, raven or crow, and skunk) differed in the fractions of the biome classes from their corresponding real animals (the fourth column of table 1). This may again reflect a small statistical power due to the small pieces of the tricksters’ data; increasing their data via enhancing the grids’ resolution revealed that the biome fractions between real and trickster animals significantly differ in 10 animals (the right column of electronic supplementary material, table S2).

Mice and rats showed exceptionally lower conditional probabilities than the other animals. Although these species appeared in certain regions where only tricksters were observed, such areas were surrounded by the regions in which real mice and rats were seen (i.e. the orange areas surrounded by blue or green areas on the world maps in figure 2).

### 3.3. Constraints by neighbour tricksters

**Figure 4.**
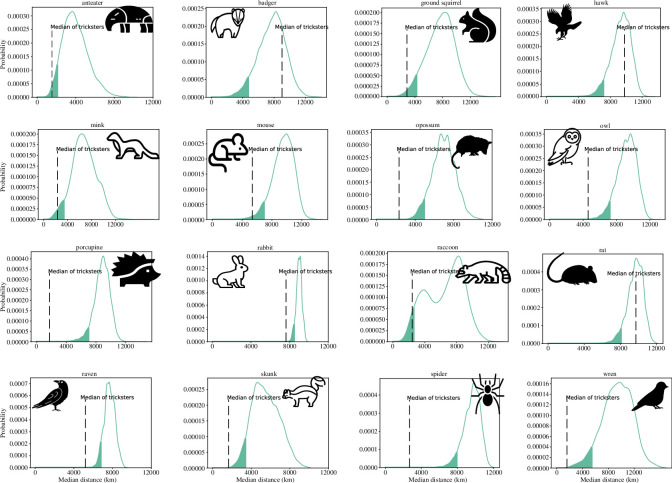
Permutation test of the distances on the world map. In the null model, the trickster animals were positioned randomly on the grid in places where the corresponding real animals were reported. For each animal, we determined the locations in which the corresponding tricksters were more densely distributed. The dashed line in every panel represents the median distance between the tricksters in the data; the curve represents the probability distribution of the median distance per the null model; and the shaded areas indicate the lowest 5% values of the distribution. The *p*-values after FDR correction have been noted in table 2.

We also investigated whether the presence of trickster animals was affected by other tricksters in the neighbourhoods (i.e. surrounding grids). The distance between societies with identical trickster animals would be shorter if these folklores were culturally transmitted from one to another than if these trickster animals were independently created in each society with a certain probability. Clusters of trickster animals are displayed on the world maps (figure 2). Potential restriction of trickster distribution within a part of biomes (the right column of electronic supplementary material, table S2) may reflect the fact that closer areas have similar climate conditions. The permutation test also revealed that the distance between the grids where trickster animals existed was shorter for 13 animals than the distance between randomly chosen grids in which the corresponding real animals existed (figure 4). These animals and the *p*-value calculated after FDR correction are noted in table 2. Increasing the resolution of the grid did not change the results of the permutation tests (electronic supplementary material, table S4). Therefore, the tricksters of a focal animal were positively affected by the presence of other tricksters in the vicinity.

**Table 2.** *P*-values in the permutation test.

category	*p*-value
anteater	$9.58\times 10^{-3}$ ^a^
badger	$7.74\times 10^{-1}$
ground squirrel	$8.20\times 10^{-3}$ ^a^
hawk	$6.12\times 10^{-1}$
mink	$1.45\times 10^{-2}$ ^a^
mouse	$7.06\times 10^{-3}$ ^a^
opossum	$9.85\times 10^{-11}$ ^a^
owl	$1.23\times 10^{-4}$ ^a^
porcupine	$1.72\times 10^{-21}$ ^a^
rabbit/hare	$1.03\times 10^{-6}$ ^a^
raccoon	$3.69\times 10^{-2}$ ^a^
rat	$4.99\times 10^{-1}$
raven/crow	$4.42\times 10^{-10}$ ^a^
skunk	$1.28\times 10^{-4}$ ^a^
spider	$6.50\times 10^{-59}$ ^a^
wren	$1.23\times 10^{-4}$ ^a^

^a^ *p*-value < 0.05 after FDR correction.

## 4. Discussion

Folklore is one of the human cultures that have the most enriched records, and the diffusion of folklore has been investigated as an example of human cultural evolution [45,59]. Because human imagination is boundless and human languages are almost unlimited in terms of expression [1], stories can contain creatures never witnessed by their tellers. Hence, fictional creatures in folklore could theoretically be shared worldwide via cultural transmission. This study, however, demonstrates that the presence of real animals is almost a prerequisite for trickster animals to appear. In other words, ecological and climatic conditions have dominant effects on contents in folklore [15–17], as on other human cultures [5–14].

This study applied a biogeographical methodology to demonstrate how certain cultural notions (in this instance, folk motifs) are limited by local ecological factors. The folklore of societies is unlikely to include focal trickster animals if the corresponding real animals were not reported there. Trickster mice and rats were exceptions; we could not, however, conclude whether the real mice and rats were missing because our data indicate only the presence, but not the absence, of the animals. For the rest of the animals, the distributions of trickster and real animals overlapped. The annual mean temperature and annual precipitation affect the distribution of many real animals. Hence, these climate conditions indirectly restrict the distribution of trickster animals in folklore (figure 1).

Figure 4 shows that the distance between reported trickster animals was closer than that when trickster animals were randomly distributed to where the corresponding real animals existed. Although such patterns would occur if the trickster folklore was culturally transmitted from the neighbourhood, other mechanisms can also produce patterns. For example, the geographically biased sampling of folklore can generate similar patterns. Alternatively, environmental conditions that Whittaker’s biome does not include may affect the distribution of tricksters. In this case, closer areas may have more similar environmental conditions. To analyse whether closer trickster folklore was culturally transmitted or not, one potential future research direction is to reconstruct the dynamics of folklore diffusion by, for example, cultural phylogenetics [60,61].

Once the diffusion of folklore is reconstructed, this would pave the way to investigate the mechanisms to generate the patterns (figures 2 and 3) observed in this study. Humans tend to focus on familiar informational content and reproduce stories as per content or schematic frameworks (i.e. schema) that they already know [62,63]. Previous experiments have shown that cognitive biases shape folklore in certain directions [62–64]. Such cognitive or behavioural processes may shape folklore incorporating trickster animals whose corresponding real animals were familiar to locals. If this is the case, we can hypothesize that the presence of real animals enhances the creation, adaptation or maintenance of corresponding trickster animals. The extinction rate of the tricksters, on the other hand, might be independent of the presence/absence of real animals because some carnivores’ tales remain in the area where the corresponding real animals have gone extinct [33–35]. Although cultural extinction has been analysed theoretically and empirically [65,66], Berezkin’s folklore database is not suitable for such analyses because dynamics of the presence/absence of folklore in each area are not available. Once the time series data of folklore and real animals are available, one can test whether the presence of real animals affects the creation/acceptance or extinction rates of trickster animals by comparing the empirical distributions of real and trickster animals with a null model that does incorporate the presence/absence of real animals. Such a null model can be built based on the dual inheritance theory that allows mismatches between environments and cultural traits [67].

One limitation of this study is that tricksters are subsets of animal folklore. Broader animal folklores can be analysed by the motif of ‘animal tales’ in Aarne Thompson Uther’s catalogue [19], although it does not provide the geographic coordinate information of folklore. There are overlaps between trickster animals and animals in the motif of ‘animal tales’, but some animals that frequently appeared only in the motif of ‘animal tales’ (see electronic supplementary material, table S1 in the study by Nakawake and Sato [19]) were not reported as tricksters. Future studies are needed to investigate whether the natural environments restrict the distribution of broader animal tales or not. More generally, future research could expand our framework to broader fictional creatures to investigate whether the contents of folklore are, in general, restricted by local environments. For example, folklore related to dragons, water-related chimeric creatures whose bodies are partially that of snakes, is described in all continents [2,31,32]. Blust [2] argues that dragons were inspired by the rainbow, a natural phenomenon worldwide. This argument would be supported by investigations of climate conditions to find correlations between dragon-related folklore and the occurrence of rainbows. One obstacle of such research would be how to determine the pairs of supernatural creatures with the motifs they are based on because the ontology of supernatural creatures can vary among literature.

The recent increase in quantitative analyses of cultural resources has advanced our understanding of human cultures by incorporating theories and methodologies employed in evolutionary biology [60,61]. Our investigation incorporates biogeographical theories and methods to explore the links between folkloristic traditions and local ecological conditions. We believe that biogeographical concepts, particularly Whittaker’s biome scheme, would enrich our understanding of the relationships between human culture and ecology. Future studies could also apply ecological approaches to move from investigating restrictions to predicting cultural distribution. Ecologists have developed statistical methods to predict the distribution of species. However, these methodologies can also apply to fictional creatures [68] and institutions [69]. Such analyses employ aspects such as climate conditions, the distribution of other species (potentially including cultures and institutions) and their interactions [70]. Furthermore, ecologists have investigated the determiners of biodiversity and temporal stability of systems [71–73], which would be applicable to investigate the stability and diversity of human culture. Collaboration with ecologists and evolutionary biologists would be promising to deepen the understanding of human culture.

## Data Availability

The original data on folklore is available from Dr Yuri Berezkin at Department of Anthropology, the European University at Saint Petersburg. The codes and derived data used in this paper are available from [74]. Electronic supplementary material is available online [75].
